# Mortality from gastroschisis in the state of Rio de Janeiro: a 10-year series

**DOI:** 10.11606/s1518-8787.2020054001757

**Published:** 2020-06-05

**Authors:** Camilla Ferreira Catarino Barreiros, Maria Auxiliadora de Souza Mendes Gomes, Saint Clair dos Santos Gomes

**Affiliations:** I Instituto de Puericultura e Pediatria Martagão Gesteira Rio de JaneiroRJ Brasil Instituto de Puericultura e Pediatria Martagão Gesteira. Rio de Janeiro, RJ, Brasil; II Instituto Nacional de Saúde da Mulher, da Criança e do Adolescente Fernandes Figueira Rio de JaneiroRJ Brasil Instituto Nacional de Saúde da Mulher, da Criança e do Adolescente Fernandes Figueira. Rio de Janeiro, RJ, Brasil

**Keywords:** Gastroschisis, mortality, Mortality, trends, Risk Factors, Health Information Systems, Tertiary Health Care, Retrospective Studies

## Abstract

**OBJECTIVE:**

To analyze mortality and associated factors in a series of gastroschisis at birth in the state of Rio de Janeiro in a 10-year period (2005 to 2014).

**METHOD:**

A retrospective cohort study, which related the databases of the Live Births Information System and the Mortality Information System by probabilistic linkage. Final database was constructed in two stages: preparation of the two initial databases and establishment of relationships between them.

**RESULTS:**

Preterm newborns and those with low birthweight had higher risk of death, with statistical significance (p = 0.03 and p = 0.006, respectively). Regarding place of birth, although death frequency was higher in maternity units than in general hospitals (p = 0.04; OR = 0.5; 95%CI 0.3–1.0), it was observed that a unit characterized as a general hospital had a high birth frequency (61.2%). Furthermore, the comparative analysis of the risk of death between this unit and others showed a 7.5 higher risk of death in general hospitals and 3.2 higher in maternity units, with statistical significance (p < 0.001). Moreover, births in level II intensive care units had 3.9 times more risk of death compared with level III (p < 0.001).

**CONCLUSION:**

This study foments the discussion of two possible strategies in the treatment of gastroschisis in newborns. First, the centralization of care in tertiary units, enabling malformation care to be analyzed in a more detailed and standardized manner. Second, and perhaps more feasible, the elaboration of clinical guidelines to standardize immediate care for gastroschisis in babies born outside tertiary centers, as well as the standardization of their transportation until arrival at the tertiary center.

## INTRODUCTION

Gastroschisis is a defect characterized by the longitudinal opening of all layers in abdominal wall. It is usually located to the right of the umbilical cord, which, in turn, is intact, without alteration in its insertion. Its differential is the externalization of the abdominal viscera, mainly intestines, without any skin or peritoneal membrane coverage^1^. Mortality in developed countries is less than 10%^2^.

In Brazil, the only official source on congenital malformations is the Live Births Information System (SINASC), by the variable 41, through which the professional describes, at the time of birth, the malformation discovered. Besides SINASC, the Mortality Information System (SIM) contributes to a better understanding of this malformation and factors that potentiate this outcome.

Considering the lack of official data on gastroschisis in Brazil, secondary data becomes a fundamental research field to understand the illness behavior in the general population. Methods such as linkage enables the development of longitudinal studies with low operational cost^3^.

Thus, the aim of this study is to analyze mortality and associated factors in a series of gastroschisis at birth in the state of Rio de Janeiro in a 10-year period (2005 to 2014) through records of children under one year old using SINASC and SIM linkage.

## METHODS

This is a retrospective cohort study, in which SINASC and SIM databases were related by the probabilistic linkage method. Final database was constructed in two stages: preparation of the two initial databases and establishment of relationships between them.

### SINASC and SIM Database Preparation

All records of the state of Rio de Janeiro in SINASC from 2005 to 2014 were used with the variable 41 (which registers the International Classification of Diseases 10th Revision [ICD-10] code for congenital malformation) filled with code Q79.3, for gastroschisis. Such criterion was defined regarding the type of malformation, visible at birth and thus easily identified^4^. Cases of multiple malformations were excluded so that deaths would not be maximized.

For SIM database development, data from 2005 to 2015 on mortality in the state of Rio de Janeiro were required. According to these data, deaths occurred in children under one year old born between 2005 and 2014, information contained in the database itself was selected. Then records whose underlying cause of death was filled out with the ICD-10 code Q79.3 were chosen. Records lacking the mother’s name and cases of fetal deaths were excluded.

### SINASC Variables on Congenital Anomaly

The Statement of Live Births (SLB) contains two fields to record congenital malformation data. The first is field 6, inserted in block I, regarding live birth identification. This field register the question “Any congenital anomalies detected?”, which possible answers are “1” when affirmative, “2” when negative, and “9” when ignored. In case of an affirmative answer, the person responsible for filling the statement should describe the identified malformation in block VI, field 41^5^.

### Relationship between SINASC and SIM Databases

Records were related by linkage technique, which enables the probabilistic relationship between two databases, aiming to detect the probability of a pair of records referring to a same individual. To do so, 3.1 OpenRecLink^6^ was used on Microsoft Windows 7.

Initially, files were standardized to minimize possible errors or spelling differences. Within SINASC database, the following variables were standardized: SLB number, mother’s name, date of birth, address and neighborhood of residence, municipality of occurrence code, health establishment code, mother’s age, mother’s marital status, mother’s educational level, number of prenatal visits, Apgar score in the first and fifth minutes, race, birthweight and sex.

Within SIM database, the following variables were standardized: SLB number, mother’s name, date of birth, address and neighborhood of residence, municipality of residence code, death certificate (DC) number, date of death, age at the time of death, race, sex and cause of death.

After standardization, the following fields were broken into components (blocking): mother’s name (soundex of the mother’s first name – FBLOCK; soundex of the mother’s last name – LBLOCK) and date of birth (month and year of birth). Thus, to relate SINASC and SIM, the blocking key composed of eight stages was used, by associating the following blocking keys: soundex of the mother’s first name (FBLOCK), soundex of the mother’s last name (LBLOCK), sex and month and year of birth.

To calculate the scores, the mother’s name and date of birth were included. They were compared using algorithms based on the Levenshtein distance. To estimate pairing parameters, Camargo Jr. and Coeli’s suggestions^7^were used.

Pairs obtained in the first blocking stage were manually reviewed by one of the researchers. For subsequent stages, and based on the manual review, pairs whose score was equal to or higher than nine were considered true positive (Box).

**Box:** Blocking stages.

Stages	Blocking strategy	Number of pairs found
Stage 1	Soundex code of the mother’s last name + Soundex code of the mother’s first name + sex + year of birth	40
Stage 2	Soundex code of mother’s first name + Soundex code of mother’s last name + sex + year of birth + month of birth	45
Stage 3	Soundex code of the mother’s first name + Soundex code of the mother’s last name + sex + year of birth	1
Stage 4	Soundex code of the mother’s first name + Soundex code of the mother’s last name + sex	0
Stage 5	Soundex code of mother’s first name + sex	6
Stage 6	Soundex code of the mother’s last name + sex	3
Stage 7	Soundex code of the mother’s first name + Soundex code of the mother’s last name	3
Stage 8	Birth year + sex	1
Total		99

To set a true positive pair, researchers used as criterion not only the mother’s name and date of birth, but also, when available, address, neighborhood, municipality and SLB number.

Death records that were not matched by the process of probabilistic linkage were manually inspected by the researcher in the original SINASC database, to ensure the software accuracy and describe the main vulnerabilities found in SINASC variable 41.

After cohort development on gastroschisis at birth records, researchers included in the study database three characteristics of the units, using place of birth and the National Health Facility Registry (NHFR) online database. First, birth unit type (general hospital or maternity unit). To classify, the team sought in NHFR information on each service characteristics. General hospitals were considered units that presented in their structure the following services: neonatal intensive care unit (NICU) level II or III, pediatric surgery unit with four beds minimum and imaging services. Such classification did not consider the NHFR information “type of establishment.” That is, even if it was a general hospital, if it did not have these services, it was considered only a maternity unit. Maternity units were characterized by the presence of NICU service and obstetrics service. Such classification was set with a health management specialist. The second characteristic was NICU presence and the third was NICU level, considering the definition established by the 2012 ordinance No. 930 of the Ministry of Health^8^.

Based on these data, birth records in private units were excluded of the analysis, as they presented different care dynamics from those of the Unified Health System (SUS).

### Statistical Analysis

SPSS^®^ was used for database preparation and data analysis. A descriptive analysis was performed based on the frequency of occurrence of the considered variables. Bivariate analysis was used to evaluate death occurrence association, and *odds ratio* (OR) and the chi-square test (*x*^2^) to evaluate the respective statistical significance (p < 0.05).

The research used SINASC and SIM data provided by Rio de Janeiro State Health Department, by means of a database license agreement and the signing of a term of responsibility. In addition, research was performed within scientific ethics standards, submitted and approved by the Research Ethics Committee (CAAE 70436717.8.0000.5269).

## RESULTS

### SINASC Frequency of Gastroschisis and Mortality

Between 2005 and 2014, in the state of Rio de Janeiro, SINASC recorded 2,213,228 live births. According to the inclusion and exclusion criteria established in this study, 769 newborns (NB) had isolated gastroschisis records: three cases for every 10,000 live births. Frequency of gastroschisis at live birth ranged from 2.7 to 4 in the 10 years studied.

After applying the inclusion and exclusion criteria in the SIM database, there were 164 records of deaths of children under one year old whose underlying cause was gastroschisis. Mortality rate was 7.4 deaths per 100,000 live births. From isolated gastroschisis birth records, 12.9% died from it. The Figure shows a historical series of the frequency of gastroschisis at birth records, mortality rate and percentage of deaths in birth records.

**Figure:**
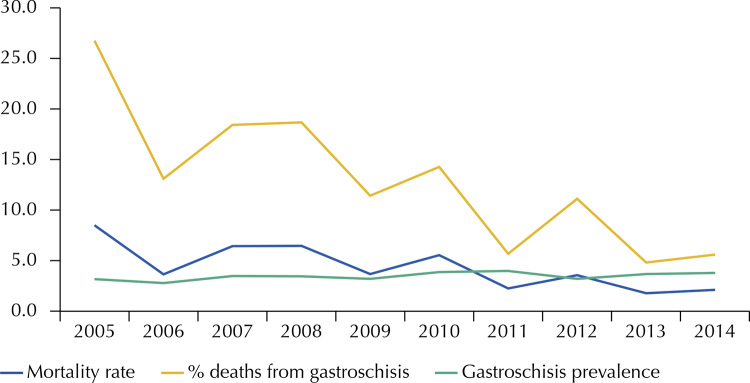
Frequency of births, percentage of death from gastroschisis and mortality rate. Ten-year historical series of Live Births Information System records and the Mortality Information System in the state of Rio de Janeiro, 2005 to 2014.

### Linkage

Through SINASC and SIM association, 99 pairs were identified. From the 65 records of deaths not located in SINASC which underlying cause was gastroschisis, field 6 of block I (identification) on malformation was blank in 3.1% (n = 2), filled with “ignored” in 6.3% (n = 4) and with “no” in 54.7% (n = 35).

Inspection process also found that in 4.7% (n = 3) of records, although identification block carried the information that the NB had congenital malformation, the anomaly was not specified in block VI, field 41. Moreover, 17.2% (n = 11) of records presented the description of a malformation other than gastroschisis, whereas 15.6% (n = 10) presented multiple anomalies.

### Final Database Description

Regarding newborns characteristics, statistical significance was found for the variables: gestational age (p = 0.03), Apgar in the first minute of life (p < 0.001), Apgar in the 5th minute of life (p = 0.002) and birthweight (p = 0.006), according to Table 1.

**Table 1:** Characteristics of records of newborns with gastroschisis in the state of Rio de Janeiro in the Live Births Information System, 2005 to 2015.

	Death				Total		p	OR (95%CI)

	Yes		No					
		
	N	%	N	%	N	%		
Sex × death								
Female	44	12.8	345	88.7	389	50.7	0.19	0.7 (0.4–1.1)
Male	55	17.0	324	85.5	379	49.3		

Total	99	14.8	669	87.1	768	100	-	-

Gestational age × death								
< 37 weeks	57	18.0	317	84.8	374	49.1	0.03	1.6 (1.04–2.4)
> 37 weeks	39	11.2	349	89.9	388	50.9		

Total	96	14.4	666	87.4	762	100.0	-	-

Apgar 1st min × death								
< 7	47	24.2	194	80.5	241	31.8	< 0.001	2.1 (1.4–3.3)
> 7	52	11.2	465	89.9	517	68.2		

Total	99	15.0	659	86.9	758	100	-	-

Apgar 5th min × death								
< 7	14	36.8	38	73.1	52	6.8	0.02	2.7 (1.4–5.1)
> 7	85	13.6	623	88.0	708	93.2		

Total	99	15.0	661	87.0	760	100.0	-	-

Birthweight × death								
< 2,500g	69	18.5	373	84.4	442	57.6	0.006	1.8 (1.1–1.9)
> 2,500g	29	9.8	296	91.1	325	42.4		
Total	98	14.6	669	87.2	767	100	-	-

OR: odds ratio; 95%CI: 95% confidence interval

In birth unit analysis, Table 2 shows that, although death proportion was lower in NB born in general hospitals compared with those born in maternity units, no statistical difference was not found.

**Table 2:** Characteristics of birth units according to the records of newborns with gastroschisis in the state of Rio de Janeiro in the Live Births Information System, 2005 to 2015.

	Death				Total		p	OR (95%CI)

	Yes		No					
		
	N	%	No	%	N	%		
Type of birth unit^a^ × death								
General Hospital	40	9.1	401	90.9	441	74.5	0.04	0.5 (0.3–1.0)
Maternity Unit	22	14.6	129	85.4	151	25.5		

Total	61	10.3	530	89.5	592	100	-	-

NICU Presence × death								
Yes	55	10.1	489	89.9	544	92.8	0.2	0.6 (0.2–1.6)
No	6	14.3	36	85.7	42	7.2		

Total	61	10.4	525	89.6	586	100	-	-

NICU Presence × death								
Level II	33	19.6	135	80.4	168	30.9	< 0.001	3.9 (2.2–6.9)
Level III	22	5.9	354	94.1	376	69.1		

Total	55	10.1	489	89.9	544	100	-	-

OR: odds ratio; 95%CI: 95% confidence interval; NICU: neonatal intensive care unit

^a^ Excluded births in private units and missing.

By this category descriptive analysis, researchers identified that a unit considered a general hospital was responsible for most births (53%). That is why the category birth unit type was analyzed in another way, by comparing the behavior of this unit with the two other categories. Table 3 shows that its death rate was much lower than the others, with statistical significance.

**Table 3:** Type of birth unit in three categories according to death, based on the records of newborns with gastroschisis in the state of Rio de Janeiro in the Live Births Information System, 2005 to 2015.

	Death				Total		p	OR (95%CI)

	Yes		No					
		
	N	%	No	%	N	%		
Three categories of birth units × death								
General Hospital	22	28.2	56	71.8	78	13.2	< 0.001	7.5 (3.7–14.9)
Maternity Unit	22	15.3	129	89.6	144	24.3	< 0.001	3.2 (1.6–6.3)
Unit A	18	5.0	345	95.0	363	61.2	-	-

Total	62	10.6	530	90.6	585	100	-	-

OR: odds ratio; 95%CI: 95% confidence interval

Death occurrence was higher in the group born in units that did not present NICU; however, there was no statistical significance. The average number of NICU beds in the state of Rio de Janeiro was 18.8, ranging from 0 to 28 beds per unit (standard-deviation [SD] = 11.3).

Regarding NICU level, death frequency was higher in NB born in a level II NICU (19.6%) than in those born in level III NICU (5.9%), with statistical significance (p < 0.001).

## DISCUSSION

In our matching process, 39.6% of death records were not found by linkage. This explains why not every birth record from 2005 to 2014 was used, but only those with reported gastroschisis in SINASC. Linkage was chosen because it is a visible malformation at birth and, theoretically, easy to detect.

A study on the correlation of SINASC field 41 identified that the correlation degree of SINASC with medical records depends on the type of malformation registered^4^. For musculoskeletal malformations, such as gastroschisis, the correlation index was very high, reaching an almost perfect adjusted Kappa. The authors concluded that this result may be related to the fact that it is a visible malformation at birth^4^. Even so, the variable is highly fragile, especially when verified that, from the unmatched cases, 54.7% were recorded in SINASC as if there was no malformation found at birth.

Nhoncanse et al.^9^ stress that this variable is not always filled by a trained professional, or even one who acknowledges medical terminologies. As there is no resolution on who should fill the SLB, it is often filled by administrative professionals. In this case, filing it in a descriptive manner will cause obstacles, disfavoring the completeness, accuracy and exactness.

In this study, frequency of gastroschisis ranged from 2.8 to 4 cases per 10,000 live births in 10 years. This last two decades increase has been reported in several studies worldwide^2,10,11^. Although the results of this study are consistent with international studies^1,12,13^ by probabilistic linkage, outcomes were underestimated.

Regarding survival rate, as per linkage final product in this study, 87.1% of gastroschisis at birth recorded in SINASC survived. By including SIM records not found in SINASC through linkage, this population’s survival rate decreases to 80.4%, quite different from high-income countries, which report over 90% of survival rate^2^. In a study conducted from a population-based of gastroschisis cases in California, only 4.6% died^10^. In low- and middle-income countries, NB have lower survival rate^11,16^ A study conducted in Jamaica showed a frequency of death from gastroschisis of 79%, sepsis being the main cause (82% of cases)^16^. In a study conducted in Uganda, death frequency was 98% of cases^17^. Whereas in Mexico, a population-based study showed that the death frequency in gastroschisis cases in the country was 32%^11^.

Gastroschisis mortality in low- and middle-income countries is primarily associated with neonatal-care-related factors, such as lack of diagnosis in prenatal care, delivery outside tertiary units, late surgery, lack of parenteral nutrition, lack of silo, lack of NICU and mechanical ventilators, crucial for the proper management of newborns with malformation, as well as factors such as prematurity and low birthweight^19^. As for high-income countries, factors that impact mortality are NB inherent and often inevitable, such as prematurity, low birthweight and the presence of complications such as atresias, perforations and intestinal necrosis^2,10^.

Nevertheless, it is noteworthy that the decrease in both gastroschisis mortality rate in the general population and the death frequency in this study. A study published in 2002, conducted at a reference center in Pernambuco, showed a 51% mortality rate^22^. Another study, also conducted in a reference center in Porto Alegre, published in 2010, showed a death frequency of 26.9%. In conclusion, although Brazil is a developing country, it has shown advances and improvements in neonatal care regarding this malformation.

As for NB characteristics, death frequency was higher in preterm than in full-term neonates. The ideal time for the birth of a NB with gastroschisis is an important field of discussion and varies greatly regarding studied outcomes^23^. Much has been debated about the early termination of pregnancy to avoid the prolonged exposure of the viscera in the amniotic fluid. Some studies note that late-planned preterm delivery is associated to lower intrauterine death rates, increased opportunity for surgical repair without the use of silo and early enteral nutrition^24,19^. On the other hand, other studies describe that full-term birth is related to shorter mechanical ventilation and parenteral nutrition, thus, shorter hospital stay^25^. In this study, prematurity increased about 1.6 times the risk of death among NB with gastroschisis. An akin study, conducted in the USA, presented similar results when using death outcome. In their findings, the lower the gestational age (GA), the higher the risk of death; however, NB on GA between 34 and 36 weeks did not present statistically significant increase (95% confidence interval [95%CI] 0.76–1.5)^2^.

Birthweight, in this study, has proven to be a risk factor for death in NB with gastroschisis. NB weighing less than 2,500g had 1.8 more risk of death when compared to those weighing 2,500g or more. This data is similar to a cohort study on population data of births and deaths by gastroschisis, conducted in the USA, in which NB weighing less than 1,500g had 7.05 (95%CI 4.16–11.95) times more risk of death, whereas NB weighing between 1,500g and 2,499g had 2.13 (95%CI 1.50–3.03) times more risk of death, both compared to NB weighing 2,500g or more (p < 0.001)^2^.

In this study, there was no significant difference for NB born in general hospitals and maternity units regarding outcome death. These two classifications aimed to identify fundamental characteristics for a health service to fully attend the NB with gastroschisis at birth. In this research, general hospitals gather the minimum necessary attributes for these cases, presenting level II or III NICU, pediatric surgery services focused on neonatal care and the enough number of beds to meet this demand, besides NICU imaging services. During data descriptive analysis stage, the researchers identified that 53% (n = 383) of cohort births occurred in a specific unit, classified as a general hospital, as it gathered all attributes. This led the researchers to analyze and compare this unit outcome death with two other typologies: general hospitals and maternity units. In this second analysis, it was identified that the unit, called Unit A, presented better results in comparison to the others. On the other hand, NB born in other general hospitals had 7.5 times more risk of death than those born in Unit A; those born in maternity units had 3.2 times more risk of death than in Unit A. The minimum attributes of general hospital category were not enough to obtain a better outcome in the group. For these data interpretation, it is important to understand that NB with gastroschisis at birth born in maternity units, due to the lack of minimal support for clinical-surgical management, are transferred to other units, especially tertiary units, improving the outcome death.

In addition, gastroschisis treatment is necessarily surgical. Abdominal wall closure may occur in more than one surgical time or, when possible, primary closure of the NB abdominal wall may be performed. In order to do so, pediatric surgery and anesthesiology team must present dexterity and important technical capacity, possibly exhibited in Unit A professionals, considering their births number.

A study conducted in California^20^ sought to compare gastroschisis care in different centers, classified as low, medium and high volume. The cut-off points for the average number of performed operations were: less than 5, from 5 to 9 and from 9 to 17 per year, respectively. The main hypothesis of this study is that gastroschisis at birth in a unit with large volumes is associated with shorter hospital stay and lower death occurrence. Regarding the study characterization of general hospitals, although presenting NHFR minimum attributes for NB with gastroschisis care, potentially, none of the units could be considered as high volume center for the malformation care but the unit analyzed separately, which presented an average volume of 76 births per year and better results. Among the remaining ones, the one with the highest number of gastroschisis at births had an average of 2.2 births per year, being considered a low-volume center.

This issue has already been discussed by the American Academy of Pediatrics^21^, which stress that potentially severe NB have a better prognosis when birth occurs in a tertiary center. Higher professional experience and the probable negative impact of the transportation both contribute to this data.

Although maternity unit births also presented a data-statistically significant risk when compared to those in Unit A, this risk was lower than in general hospitals. It is possible that the risk of births in low-volume centers with little expertise excels the probable risk of a tertiary center transfer after birth, as in maternity unit births. Yet, a limitation should be considered: as it is a SINASC and SIM analysis, there is no “surgical procedure site” or “transfer site” variable, enabling only new hypotheses raise.

In this study, birth occurrence in level II NICU increased the chance of death by 3.9 times. According to 2012 Ordinance No. 930, the difference between the level II and III NICU is that the latter, besides all the characteristics of the first, requires: minimum 50% of on-call workers to be certifiably qualified neonatologists or to have a title in pediatric intensive care medicine; a coordinating nurse with a specialization degree in intensive care/neonatal intensive care or at least five years of certified professional experience in the area; one shift nurse, unit-exclusive, for every five beds or fraction; a physiotherapy coordinator with a specialization degree in pediatric or neonatal intensive care or in another specialty severe-patient-care related; four infusion pump per bed or fraction and a microprocessor-based mechanical ventilation for each bed.

The ordinance shows that technical assistance is higher in level III NICU, justifying a possible reduction in NB mortality when compared to level II NICU births. In Brazil, however, this classification is not properly used because it is not within clinical scope. That is, for specialists, it does not reflect a relevant difference. Moreover, level III NICU importance, in this analysis, may be disguised due to Unit A NHFR classification as a level III NICU. Thus, what may have interfered in this result was Unit A good performance in the clinical-surgical management of gastroschisis.

## CONCLUSION

The quality of SINASC records on gastroschisis may have influenced this study results or even underestimated the outcome death. NB with GA less than 37 weeks, birthweight under 2,500g and Apgar scale in the first and fifth minutes had a higher risk of outcome death (p = 0.003, p = 0.006, p < 0.001 and p = 0.02, respectively). Being born outside a large-volume center increased 5.1 times (p < 0.001) the risk of death; whereas out of units with level III NICU profile increased it by 3.8 times (p < 0.001).

This study foments the discussion of two possible strategies in the treatment of gastroschisis in NB. First, the centralization of care in tertiary units, enabling malformation care to be analyzed in a more detailed and standardized manner. The second, and perhaps more feasible, would be the elaboration of clinical guidelines that standardize immediate care to newborns with gastroschisis born outside tertiary centers, as well as the standardization of their transport until arrival at the tertiary center, minimizing complications caused by inadequate after birth management.
