# Supplementary material for: Mortality from gastroschisis in the state of Rio de Janeiro: a 10-year series
Source: Rev Saude Publica. 2020 Jun 5;54:63. doi: 10.11606/s1518-8787.2020054001757 (PMC7274210; doi:10.11606/s1518-8787.2020054001757)
Supplement: Translation

# Translation: Mortalidade por gastrosquise no estado do Rio de Janeiro: uma série de 10 anos

**Authors:** Camilla Ferreira Catarino Barreiros, Maria Auxiliadora de Souza Mendes Gomes, Saint Clair dos Santos Gomes

**Affiliations:** I Brasil Instituto de Puericultura e Pediatria Martagão Gesteira. Rio de Janeiro, RJ, Brasil; II Brasil Instituto Nacional de Saúde da Mulher, da Criança e do Adolescente Fernandes Figueira. Rio de Janeiro, RJ, Brasil

**Keywords:** Gastrosquise, mortalidade, Mortalidade, tendências, Fatores de Risco, Sistemas de Informação em Saúde, Atenção Terciária à Saúde, Estudos Retrospectivos

## Abstract

**OBJETIVO:**

Analisar a mortalidade e fatores associados em uma série de nascimentos com gastrosquise no estado do Rio de Janeiro em 10 anos (2005 a 2014).

**MÉTODO:**

Estudo de coorte retrospectiva, no qual foram relacionadas as bases de dados do Sistema de Informação sobre Nascidos Vivos e do Sistema de Informação sobre Mortalidade por *linkage* probabilístico. A base de dados final foi construída em duas etapas, que consistiram em: preparo das duas bases de dados iniciais e estabelecimento de relações entre elas.

**RESULTADOS:**

Os recém-nascidos pré-termo e os com baixo peso ao nascer tiveram maior chance de óbito, com significância estatística (p = 0,03 e p = 0,006, respectivamente). Em relação ao local de nascimento, embora a frequência de óbito tenha sido maior nas maternidades do que em hospitais gerais (p = 0,04; OR = 0,5; IC95% 0,3–1,0), foi observado que uma unidade caracterizada como hospital geral apresentou uma frequência alta de nascimentos (61,2%) e, na análise comparativa da chance de óbito dessa unidade com as demais, encontrou-se uma chance de morrer 7,5 maior em hospitais gerais e 3,2 maior em maternidades, com significância estatística (p < 0,001). Além disso, nascer em unidades de terapia intensiva tipo II aumentou a chance de óbito em 3,9 vezes em comparação com as do tipo III (p < 0,001).

**CONCLUSÃO:**

Este estudo dá subsídios para a discussão de duas possíveis estratégias no tratamento de recém-nascidos com gastrosquise. A primeira seria a centralização do cuidado em unidades terciárias, possibilitando que o cuidado à malformação seja analisado de forma mais minuciosa e padronizada. A segunda, e talvez mais factível, seria a elaboração de diretrizes clínicas que padronizem o cuidado imediato aos bebês com gastrosquise nascidos fora de centros terciários, bem como a padronização do transporte deles até a chegada ao centro terciário.

## INTRODUÇÃO

A gastrosquise é um defeito caracterizado pela abertura longitudinal de todas as camadas da parede abdominal. Habitualmente está localizada à direita do cordão umbilical, que por sua vez apresenta-se íntegro sem alteração em sua inserção. Seu diferencial está na exteriorização das vísceras abdominais, principalmente intestinos, sem qualquer cobertura de pele ou membrana peritoneal^1^. A mortalidade em países desenvolvidos é menor que 10%^2^.

No Brasil a única fonte de informação oficial sobre malformações congênitas é o Sistema de Informação sobre Nascidos Vivos (Sinasc), pela variável de número 41, na qual o profissional descreve, no momento do nascimento, a malformação encontrada. Além do Sinasc, o Sistema de Informação sobre Mortalidade (SIM) também colabora para um melhor entendimento dessa malformação e de quais os fatores que potencializam esse desfecho.

Considerando a ausência de dados oficiais sobre gastrosquise no Brasil, o uso de dados secundários torna-se um campo de pesquisa fundamental para compreender o comportamento do agravo na população geral. O uso de métodos como *linkage*, por exemplo, possibilita a construção de estudos longitudinais com baixo custo operacional^3^.

Sendo assim, o objetivo deste estudo é analisar a mortalidade e fatores associados em uma série de nascimentos com gastrosquise no estado do Rio de Janeiro em 10 anos (2005 a 2014) construída por registros de crianças com menos de um ano de idade mediante *linkage* entre o Sinasc e o SIM.

## MÉTODOS

Trata-se de um estudo de coorte retrospectiva, no qual foram relacionadas as bases de dados do Sinasc e do SIM pelo método de *linkage* probabilístico. A base final de dados foi construída em duas etapas: preparo das bases de dados iniciais e estabelecimento de relações entre essas bases de dados.

### Preparo do Banco de Dados do Sinasc e do SIM

Foram incluídos todos os registros do estado do Rio de Janeiro no Sinasc de 2005 a 2014 com o campo 41 (que registra os códigos da 10ª revisão da Classificação Internacional de Doenças [CID-10] para malformação congênita) preenchido com o código Q79.3, referente à gastrosquise. Este critério foi definido com base no tipo de malformação estudada, visível ao nascimento e, portanto, facilmente identificada^4^. Foram excluídos os casos de malformações múltiplas, com o objetivo de não potencializar o desfecho óbito.

Para a construção da base de dados do SIM, foi solicitado o banco de dados sobre mortalidade dos anos de 2005 a 2015, no estado do Rio de Janeiro. A partir desses dados foram selecionados os óbitos ocorridos em menores de um ano nascidos entre 2005 e 2014, informação contida no próprio banco. Depois foram recrutados os registros cuja causa básica do óbito foi preenchida com o código CID-10 Q79.3. Foram excluídos os registros sem preenchimento do nome da mãe e os casos de óbitos fetais.

### Variáveis sobre Anomalia Congênita no Sinasc

Na declaração de nascido vivo (DNV) existem dois campos que registram a informação referente à malformação congênita. O primeiro é o campo 6, inserido no bloco I, referente à identificação do nascido vivo. Esse campo registra a pergunta “Detectada alguma anomalia congênita?”, sendo as opções de resposta “1” quando afirmativa, “2” quando negativa e “9” para ignorada. Em caso de resposta afirmativa, o responsável pelo preenchimento deverá descrever a malformação identificada no campo 41 do Bloco VI^5^.

### Relacionamento entre as Bases de Dados do Sinasc e do SIM

Os registros foram relacionados a partir da técnica de *linkage,* que possibilita o relacionamento probabilístico entre duas bases de dados, objetivando detectar a probabilidade de que um par de registros se refira a um mesmo indivíduo. Para tal, utilizou-se o *software* OpenRecLink versão 3.1^6^ na plataforma Microsoft Windows 7.

Inicialmente foi realizada a padronização dos arquivos, visando minimizar possíveis erros ou diferenças de grafia. No banco do Sinasc, foram padronizadas as seguintes variáveis: número da DNV, nome da mãe, data de nascimento, endereço e bairro de residência, código do município de ocorrência, código do estabelecimento de saúde, idade da mãe, estado civil da mãe, escolaridade da mãe, número de consultas no pré-natal, escala de Apgar no primeiro e quinto minutos, raça, peso ao nascer e sexo.

No banco de dados do SIM foram padronizadas as seguintes variáveis: número da DNV, nome da mãe, data de nascimento, endereço e bairro de residência, código do município de residência, número da declaração de óbito (DO), data do óbito, idade no momento do óbito, raça, sexo e causa do óbito.

Após a padronização, realizaram-se as quebras em componentes (blocagem) dos campos nome da mãe (*soundex* do primeiro nome da mãe – PBLOCO; *soundex* do último nome da mãe – UBLOCO) e data de nascimento (mês de nascimento e ano de nascimento). Assim, para o relacionamento do Sinasc com o SIM, se utilizou a chave de blocagem composta por oito passos, por meio da associação das seguintes chaves de blocagem: *soundex* do primeiro nome da mãe (PBLOCO), *soundex* do último nome da mãe (UBLOCO), sexo, mês de nascimento e ano de nascimento.

Para o cálculo dos escores, empregaram-se os campos nome da mãe e data de nascimento, que foram comparados utilizando algoritmos baseados na distância Levenshtein. Para a estimativa dos parâmetros de pareamento, foram utilizados os sugeridos por Camargo Jr. e Coeli^7^.

Todos os pares obtidos no primeiro passo de blocagem foram revisados manualmente por um dos pesquisadores. Nos passos subsequentes, com base na revisão manual, foram considerados verdadeiros positivos aqueles cujo escore foi igual ou superior a nove (Quadro).

**Quadro:** Passos de blocagem.

Passos	Estratégia de blocagem	N° de pares encontrados
Passo 1	Código Soundex do último nome da mãe + código Soundex do primeiro nome da mãe + sexo + ano de nascimento	40
Passo 2	Código Soundex do primeiro nome da mãe + código Soundex do último nome da mãe + sexo + ano de nascimento + mês de nascimento	45
Passo 3	Código Soundex do primeiro nome da mãe + código Soundex do último nome da mãe + sexo + ano de nascimento	1
Passo 4	Código Soundex do primeiro nome da mãe + código Soundex do último nome da mãe + sexo	0
Passo 5	Código Soundex do primeiro nome da mãe + sexo	6
Passo 6	Código Soundex do último nome da mãe + sexo	3
Passo 7	Código Soundex do primeiro nome da mãe + Código Soundex do último nome da mãe	3
Passo 8	Ano nascimento + sexo	1
Total		99

Para o estabelecimento de um par verdadeiro positivo, os pesquisadores utilizaram como critério não apenas o nome da mãe e a data de nascimento, mas também informações como endereço, bairro, município e número da DNV, quando disponíveis.

Ressalta-se que os registros de óbitos não pareados pelo processo de *linkage* probabilístico foram inspecionados de forma manual pela pesquisadora na base original do Sinasc, para garantir a precisão da utilização do *software*, bem como descrever as principais fragilidades encontradas na variável 41 do Sinasc.

Após a construção da coorte de registros de nascimentos com gastrosquise, os pesquisadores, utilizando a variável local de nascimento e o banco de dados on-line do Cadastro Nacional de Estabelecimentos de Saúde (CNES), incluíram no banco de dados do estudo três características das unidades. A primeira foi o tipo de unidade do nascimento (hospital geral ou maternidade). Para essa classificação, a equipe buscou no CNES informações sobre as características de cada serviço, sendo consideradas hospitais gerais as unidades que apresentavam em sua estrutura os seguintes serviços: unidade de terapia intensiva neonatal (UTIN) tipo II ou tipo III, unidade de cirurgia pediátrica com no mínimo quatro leitos e serviços de imagem. Esta classificação foi realizada independentemente da informação “tipo de estabelecimento” contida no CNES, ou seja, mesmo que se tratasse de uma unidade de hospital geral, se não tivesse os serviços listados acima, o local foi considerado apenas maternidade. As maternidades foram caracterizadas pela presença de serviço de UTIN e serviço de obstetrícia. Esta classificação foi definida com uma especialista na área de gestão em saúde. A segunda característica foi a presença de UTIN e a terceira o tipo de UTIN, considerando a definição estabelecida pelo Ministério da Saúde em sua portaria nº 930 de 2012^8^.

A partir desses dados, para a análise foram excluídos os registros de nascimentos ocorridos em unidades privadas, por apresentarem dinâmica assistencial diferente das unidades do Sistema Único de Saúde (SUS).

### Analise Estatística

O SPSS^®^ foi utilizado para a etapa de preparação dos bancos e análise dos dados. Foi realizada uma análise descritiva a partir das frequências de ocorrência das variáveis consideradas. Foram utilizados a análise bivariada para avaliar a associação entre a ocorrência do óbito, o *odds ratio* (OR) e o teste do qui-quadrado (*x*^2^) para hipótese com cálculo da respectiva significância estatística (p < 0,05).

A pesquisa utilizou dados do Sinasc e do SIM fornecidos pela Secretaria Estadual de Saúde do estado do Rio de Janeiro, mediante termo de cessão e utilização dos bancos de dados e assinatura do termo de responsabilidade. Além disso, foi realizada dentro dos padrões da ética científica, tendo sido submetida e aprovada pelo Comitê de Ética em Pesquisa (registro no CAAE 70436717.8.0000.5269).

## RESULTADOS

### Frequência da Gastrosquise no Sinasc e Mortalidade

No período entre 2005 a 2014, no estado do Rio de Janeiro, foram registrados no Sinasc 2.213.228 nascidos vivos. De acordo com os critérios de inclusão e exclusão estabelecidos neste estudo, 769 recém-nascidos (RN) apresentavam registro de gastrosquise isolada no Sinasc, compreendendo três casos para cada 10.000 nascidos vivos. A frequência variou entre 2,7 e 4 nascidos vivos com gastrosquise nos 10 anos estudados.

Após a aplicação dos critérios de inclusão e exclusão no banco de dados do SIM, restaram 164 registros de óbitos de menores de um ano cuja causa básica foi gastrosquise. A taxa de mortalidade foi de 7,4 óbitos para cada 100.000 nascidos vivos. Dos registros de nascimento com gastrosquise isolada, 12,9% foram a óbito por essa causa. Na Figura visualiza-se uma série histórica da frequência dos registros dos nascimentos com gastrosquise, taxa de mortalidade e percentual de óbitos nos registros de nascimento.

**Figura:** Frequência de nascimentos, percentual de óbito por gastrosquise e taxa de mortalidade. Série histórica de 10 anos dos registros do Sistema de Informação sobre Nascidos Vivos e do Sistema de Informação sobre Mortalidade no estado do Rio de Janeiro, 2005 a 2014.

#### Linkage

Identificaram-se 99 pares na associação do Sinasc com o SIM. Dos 65 registros de óbitos com causa básica gastrosquise não localizados no Sinasc, o campo 6 do bloco I (identificação), sobre a malformação, estava em branco em 3,1% (n = 2), preenchido com “ignorado” em 6,3% (n = 4) e com “não” em 54,7% (n = 35).

Ainda no processo de inspeção, verificou-se que, em 4,7% (n = 3) dos registros, embora no bloco de identificação houvesse a afirmação de que o RN apresentava malformação congênita, não havia a especificação de qual a anomalia no bloco VI, campo 41. Além disso, em 17,2% (n = 11) dos registros, havia descrição de outra malformação e não da gastrosquise, enquanto em 15,6% (n = 10) constavam múltiplas anomalias.

## Descrição da Base de Dados Final

Sobre as características dos RN, houve significância estatística para as variáveis: idade gestacional (p = 0,03), Apgar no primeiro minuto de vida (p < 0,001), Apgar no 5º minuto de vida (p = 0,002) e peso ao nascer (p = 0,006), conforme a Tabela 1.

**Tabela 1:** Característica dos registros de recém-nascidos com gastrosquise no estado do Rio de Janeiro no Sistema de Informação sobre Nascidos Vivos, 2005 a 2015.

	Óbito				Total		p	OR (IC95%)

	Sim		Não					
		
	N	%	N	%	N	%		
Sexo × óbito								
Feminino	44	12,8	345	88,7	389	50,7	0,19	0,7 (0,4–1,1)
Masculino	55	17,0	324	85,5	379	49,3		
Total	99	14,8	669	87,1	768	100	-	-
Idade gestacional × óbito								
< 37 semanas	57	18,0	317	84,8	374	49,1	0,03	1,6 (1,04–2,4)
> 37 semanas	39	11,2	349	89,9	388	50,9		
Total	96	14,4	666	87,4	762	100,0	-	-
Apgar 1º min × óbito								
< 7	47	24,2	194	80,5	241	31,8	< 0,001	2,1 (1,4–3,3)
> 7	52	11,2	465	89,9	517	68,2		
Total	99	15,0	659	86,9	758	100	-	-
Apgar 5º min × óbito								
< 7	14	36,8	38	73,1	52	6,8	0,02	2,7 (1,4–5,1)
> 7	85	13,6	623	88,0	708	93,2		
Total	99	15,0	661	87,0	760	100,0	-	-
Peso ao nascer × óbito								
< 2.500g	69	18,5	373	84,4	442	57,6	0,006	1,8 (1,1–1,9)
> 2.500g	29	9,8	296	91,1	325	42,4		
Total	98	14,6	669	87,2	767	100	-	-

OR: *odds ratio*; IC95%: intervalo de confiança de 95%

Na análise das unidades de nascimento, observa-se na Tabela 2 que, embora a proporção de óbito tenha sido menor no grupo de RN que nasceram em unidades do tipo hospital geral em relação aos que nasceram em maternidade, não houve diferença estatística.

**Tabela 2:** Características das unidades de nascimento de acordo com os registros de recém-nascidos com gastrosquise no estado do Rio de Janeiro no Sistema de Informação sobre Nascidos Vivos, 2005 a 2015.

	Óbito				Total		p	OR (IC95%)

	Sim		Não					
		
	N	%	Não	%	N	%		
Tipo de unidade de nascimento^a^ × óbito								
Hospital geral	40	9,1	401	90,9	441	74,5	0,04	0,5 (0,3–1,0)
Maternidade	22	14,6	129	85,4	151	25,5		
Total	61	10,3	530	89,5	592	100	-	-
Presença de UTIN × óbito								
Sim	55	10,1	489	89,9	544	92,8	0,2	0,6 (0,2–1,6)
Não	6	14,3	36	85,7	42	7,2		
Total	61	10,4	525	89,6	586	100	-	-
Tipo de UTIN × óbito								
Tipo II	33	19,6	135	80,4	168	30,9	< 0,001	3,9 (2,2–6,9)
Tipo III	22	5,9	354	94,1	376	69,1		
Total	55	10,1	489	89,9	544	100	-	-

OR: *odds ratio*; IC95%: intervalo de confiança de 95%; UTIN: unidade de terapia intensiva neonatal

^a^ Excluídos nascimentos em unidades privadas e *missing*.

Na análise descritiva dessa categoria, os pesquisadores identificaram que uma unidade tipificada como hospital geral foi responsável pela maior parte dos nascimentos (53%), motivo pelo qual se achou necessário analisar a categoria tipo de unidade de outra forma, comparando o comportamento dessa unidade com as duas outras categorias. Na Tabela 3 pode-se observar que sua proporção de óbito foi bem inferior às demais, com significância estatística.

**Tabela 3:** Tipo de unidade de nascimento em três categorias segundo óbito, de acordo com os registros de recém-nascidos com gastrosquise no estado do Rio de Janeiro no Sistema de Informação sobre Nascidos Vivos, 2005 a 2015.

	Óbito				Total		p	OR (IC95%)

	Sim		Não					
		
	N	%	Não	%	N	%		
Tipo de unidade de nascimento com três categorias × óbito								
Hospital geral	22	28,2	56	71,8	78	13,2	< 0,001	7,5 (3,7–14,9)
Maternidade	22	15,3	129	89,6	144	24,3	< 0,001	3,2 (1,6–6,3)
Unidade A	18	5,0	345	95,0	363	61,2	-	-

Total	62	10,6	530	90,6	585	100	-	-

OR: *odds ratio*; IC95%: intervalo de confiança de 95%

A ocorrência de óbitos foi maior no grupo que nasceu em unidades que não apresentavam UTIN; no entanto, não houve significância estatística. A média de número de leitos de UTIN no estado do Rio de Janeiro foi de 18,8, variando de 0 a 28 leitos por unidade (desvio-padrão [DP] = 11,3).

No que se refere ao tipo de UTIN, a frequência de óbitos foi maior no grupo dos RN que nasceram em UTIN do tipo II (19,6%) em relação aos que nasceram em UTIN do tipo III (5,9%), com significância estatística (p < 0,001).

## DISCUSSÃO

No processo de pareamento deste estudo, 39,6% dos registros de óbitos não foram encontrados no *linkage*. Isso se explica porque não foram utilizados todos os registros de nascimento no período de 2005 a 2014, mas sim os com presença de gastrosquise informada no Sinasc. Optou-se pela realização do *linkage* dessa forma por se tratar de uma malformação visível ao nascimento e, em teoria, de fácil notificação.

Em um estudo sobre concordância do campo 41 do Sinasc, identificou-se que o grau de concordância do Sinasc com prontuários depende do tipo de malformação registrada^4^. Para malformações do aparelho osteomuscular, como a gastrosquise, o índice de concordância foi bem alto e chegou a um Kappa ajustado quase perfeito. Os autores concluíram que esse resultado pode estar atrelado ao fato de se tratar de uma malformação visível ao nascimento^4^. Mesmo assim, observa-se uma fragilidade grande da variável, principalmente ao verificar que, dos casos não pareados, 54,7% dos registros constavam no Sinasc como se não houvesse malformação encontrada no momento do nascimento.

Nhoncanse et al.^9^ destacam que nem sempre o preenchimento dessa variável ocorre por profissional capacitado, ou mesmo profissional que tenha afinidade com terminologias médicas. Por não haver uma determinação sobre quem deve preencher a DNV, muitas vezes ela acaba sendo preenchida por profissionais administrativos e, nesse caso, o preenchimento de forma descritiva terá entraves, não favorecendo a completeza, a acurácia e a precisão da variável.

Neste estudo a frequência de gastrosquise variou, em 10 anos, de 2,8 a 4 casos por 10.000 nascidos vivos. Esse aumento nas últimas duas décadas tem sido relatado em diversos estudos por todo o mundo^2,10,11^. Ainda que os resultados deste estudo sejam compatíveis com estudos internacionais^1,12,13^ pelo *linkage* probabilístico, percebe-se que a mesma foi subestimada.

Sobre a taxa de sobrevivência, de acordo com o produto final do *linkage*, neste estudo 87,1% dos registros de nascimento com gastrosquise no Sinasc sobreviveram. Incluindo os registros do SIM que não foram encontrados no Sinasc por meio do *linkage*, a taxa de sobrevivência para essa população diminuiria para 80,4%, bem diferente de países de alta renda, que referem uma taxa de sobrevivência maior que 90%^2^. Na Califórnia, em um estudo realizado a partir de uma base populacional de casos de gastrosquise, apenas 4,6% foram a óbito^10^. Em países de baixa e média renda, esses RN têm menor sobrevida^11,16^. Um estudo realizado na Jamaica revelou uma frequência de óbito por gastrosquise de 79%, sendo a sepse a principal causa (82% dos casos)^16^. Em outro, realizado na Uganda, a frequência de óbito foi de 98% dos casos^17^. Já no México, um estudo de base populacional demonstrou que a frequência de óbito em casos de gastrosquise no país foi de 32%^11^.

A mortalidade por gastrosquise em países de baixa e média renda está associada principalmente a fatores ligados à assistência neonatal, como ausência de diagnóstico no pré-natal, parto fora de centros terciários, cirurgia tardia, ausência de nutrição parenteral, ausência de silo, ausência de UTIN e ventiladores mecânicos que são cruciais para o manejo adequado do RN com a malformação, além de fatores como prematuridade e baixo peso ao nascer^19^. Já em países de alta renda, os fatores que impactam a mortalidade são inerentes ao RN e muitas vezes inevitáveis, como a prematuridade, baixo peso ao nascer e presença de complicações como atresias, perfurações e necroses intestinais^2,10^.

Ainda assim, há de se considerar a notória queda tanto da taxa de mortalidade por gastrosquise na população geral quanto da frequência de óbitos neste estudo. Um estudo publicado em 2002, realizado em um centro de referência em Pernambuco, apresentou uma frequência de mortalidade de 51%^22^. Outro realizado também em um serviço de referência em Porto Alegre, publicado em 2010, apresentou uma frequência de óbitos de 26,9%. Percebe-se que, mesmo tratando-se de um país em desenvolvimento, o Brasil tem demonstrado avanços e melhoras na assistência neonatal para essa malformação.

Sobre as características dos RN, a frequência de óbito foi maior nos pré-termo que nos nascidos a termo. O momento ideal para o nascimento de um RN com gastrosquise é um importante campo de discussão e varia muito em relação aos desfechos estudados^23^. Muito se debate sobre a interrupção precoce da gestação a fim de evitar exposição prolongada das vísceras no líquido amniótico, e alguns estudos observam que o parto prematuro tardio planejado está relacionado às menores taxas de óbito intraútero, aumento da oportunidade do reparo cirúrgico sem a utilização de silo e início precoce de nutrição enteral^24,19^. Por outro lado, outros estudos descrevem que o nascimento a termo está relacionado ao menor tempo de ventilação mecânica e de nutrição parenteral, logo, ao menor tempo de internação^25^. Neste estudo a prematuridade aumentou em 1,6 vezes a chance de morrer entre os RN com gastrosquise. Um estudo semelhante a este, desenvolvido nos EUA, apresentou resultados semelhantes ao utilizar o desfecho óbito. Em seus achados, quanto menor a idade gestacional (IG), maior o risco de óbito; no entanto, os RN com IG entre 34 e 36 semanas não apresentaram aumento com significância estatística (intervalo de confiança de 95% [IC95%] 0,76–1,5)^2^.

O peso ao nascer, neste estudo, demonstrou ser um fator de risco para o óbito em RN com gastrosquise. Os RN com menos de 2.500 g apresentaram 1,8 vezes mais chances de morrer quando comparados aos com 2.500g ou mais. Esse dado é semelhante a um estudo de coorte de dados populacionais, de nascimentos e óbitos por gastrosquise, desenvolvido nos EUA, no qual o grupo de RN com peso ao nascer menor que 1.500 g apresentou 7,05 (IC95% 4,16–11,95) vezes mais chances de morrer, enquanto os RN com peso entre 1.500 g e 2.499 g apresentaram 2,13 (IC95% 1,50–3,03) vezes mais chances de morrer, ambos comparados aos RN com 2.500 g ou mais (p < 0,001)^2^.

Neste estudo não houve diferença significativa para os RN nascidos em hospitais gerais e maternidades em relação ao desfecho óbito. A classificação dessas duas categorias objetivou identificar características fundamentais para que um serviço de saúde atenda de forma plena o RN com gastrosquise desde o nascimento. Nesta pesquisa, o hospital geral reúne atributos mínimos necessários para esses casos, por apresentar suportes de UTIN tipo II ou III e serviços de cirurgia pediátrica com foco neonatal e número de leitos suficientes para suprir essa demanda, além de serviços de imagem. Na fase da análise descritiva desses dados, os pesquisadores identificaram que 53% (n = 383) dos nascimentos da coorte ocorreram em uma unidade específica, classificada como hospital geral por reunir todos os atributos supracitados. Esse motivo direcionou os pesquisadores a analisar e comparar o desfecho óbito dessa unidade com as outras duas tipologias: hospitais gerais e maternidades. Nessa segunda análise foi possível identificar que a unidade, denominada Unidade A, apresentou melhores resultados em relação às demais. Por outro lado, os RN que nasceram em outros hospitais gerais apresentaram 7,5 vezes mais chances de morrer que quem nasceu na Unidade A, enquanto os nascidos em maternidades tiveram 3,2 mais chances de morrer que na Unidade A. Os atributos mínimos da categoria hospital geral não foram suficientes para obtenção de melhor desfecho no grupo. Para a interpretação desse dado, é importante entender que os RN que nasceram com gastrosquise em maternidades, por não apresentarem suporte mínimo para o manejo clínico-cirúrgico, são transferidos para outras unidades, em especial centros terciários, melhorando o desfecho óbito.

Além disso, o tratamento da gastrosquise é necessariamente cirúrgico. O fechamento da parede abdominal pode ocorrer em mais de um tempo cirúrgico ou, quando possível, pode ser realizado fechamento primário da parede abdominal do RN. Para isso é necessária destreza e importante capacidade técnica por parte das equipes de cirurgia pediátrica e anestesiologia, características possivelmente presentes na Unidade A, considerando seu número de nascimentos.

Um estudo realizado na Califórnia^20^ buscou comparar o atendimento por gastrosquise em diferentes centros, que foram classificados entre baixo, médio e alto volume. Os pontos de corte para o número médio de operações realizadas foram menos que 5, de 5 a 9 e de 9 a 17 por ano, respectivamente. A hipótese principal desse estudo é que o nascimento com gastrosquise em centro de grandes volumes está associado a menor tempo de internação e menor número de óbitos. Sobre a caracterização dos hospitais gerais do estudo, embora apresentem atributos mínimos disponíveis no CNES para o atendimento ao RN com gastrosquise, existe a hipótese de que nenhuma das unidades poderia ser considerada como um centro de grande volume do atendimento à malformação, exceto na unidade analisada separadamente, que apresentou um volume médio de nascimentos de 76 por ano e melhores resultados. Dentre as restantes, a que apresentou maior número de nascimentos com gastrosquise teve em média 2,2 nascimentos por ano, ou seja, pode ser considerada um centro de baixo volume de casos.

Essa questão já é discutida pela Academia Americana de Pediatria^21^, que destaca que RN potencialmente graves têm um melhor prognóstico quando o nascimento ocorre em centros terciários. Fatores que contribuem para este dado são o aumento da experiência dos profissionais e o potencial efeito negativo do processo de transporte.

Embora os nascimentos ocorridos em maternidades também tenham apresentado risco com significância estatística dos dados quando comparados aos da Unidade A, esse risco foi menor em relação aos de hospitais gerais. É possível que o risco do nascimento em centros de baixo volume, com pouca expertise, se sobreponha ao risco potencial de uma transferência para um hospital terciário após o nascimento, como no caso dos nascimentos ocorridos em maternidades. Contudo, há de se considerar uma limitação: por se tratar de uma análise de Sinasc e SIM, não existe a variável “local do procedimento cirúrgico” nem “local de transferência”, possibilitando apenas o levantamento de novas hipóteses.

Neste estudo a ocorrência do nascimento em UTIN tipo II aumentou a chance de óbito em 3,9 vezes. De acordo com a portaria nº 930 de 2012, a diferença entre a UTIN tipo II para a tipo III é que na última, além de todas as características da primeira, é necessário ter: no mínimo 50% dos plantonistas com certificado de habilitação em neonatologia ou título em medicina intensiva pediátrica; um enfermeiro coordenador com título de especialização em terapia intensiva/terapia intensiva neonatal ou no mínimo cinco anos de experiência profissional comprovada de atuação na área; um enfermeiro plantonista assistencial por turno, exclusivo da unidade, para cada cinco leitos ou fração; um coordenador de fisioterapia com título de especialização em terapia intensiva pediátrica ou neonatal ou em outra especialidade relacionada à assistência ao paciente grave; quatro bombas de infusão por leito ou fração e um ventilador mecânico microprocessado para cada leito.

Pela portaria observa-se que a assistência técnica é maior na UTIN tipo III, justificando uma possível redução da mortalidade nesse grupo quando comparado ao que nasceu em UTIN tipo II. No entanto, há de se considerar que no Brasil esta classificação não é bem utilizada, porque não está no escopo clínico, ou seja, não reflete, para os especialistas, uma diferença importante. Além disso, o grande peso da UTIN tipo III, nesta análise, pode estar mascarada pelo fato de a Unidade A estar classificada no CNES como UTIN tipo III; portanto, o que possivelmente interferiu nesse resultado, foi o bom desempenho da Unidade A no manejo clínico-cirúrgico da gastrosquise.

## CONCLUSÃO

A qualidade do registro da gastrosquise no Sinasc pode ter influenciado nos resultados desse estudo ou mesmo subestimado o desfecho óbito. Os RN com IG menor que 37 semanas, peso ao nascer inferior a 2.500 g e escala de Apgar no primeiro e quinto minutos apresentaram maior chance do desfecho óbito (p = 0,003, p = 0,006, p < 0,001 e p = 0,02, respectivamente). Nascer fora de um centro de grande volume de cirurgias e de unidades com perfil de UTIN tipo III aumentou a chance de morrer em 5,1 vezes (p < 0,001) e 3,8 vezes (p < 0,001), respectivamente.

Este estudo dá subsídios para a discussão de duas possíveis estratégias no tratamento ao RN com gastrosquise. A primeira seria a centralização do cuidado aos RN com gastrosquise em unidades terciárias, possibilitando que o cuidado à malformação seja analisado de forma mais minuciosa e padronizada. A segunda, e talvez mais factível, seria a elaboração de diretrizes clínicas que padronizem o cuidado imediato aos RN com gastrosquise nascidos fora de centros terciários, bem como a padronização do transporte do RN até a chegada no centro terciário, minimizando possíveis complicações favorecidas pelo manejo inadequado após o nascimento.
